# Hypoxia and glucose metabolism assessed by FMISO and FDG PET for predicting IDH1 mutation and 1p/19q codeletion status in newly diagnosed malignant gliomas

**DOI:** 10.1186/s13550-021-00806-6

**Published:** 2021-07-21

**Authors:** Kenta Suzuki, Nobuyuki Kawai, Tomoya Ogawa, Keisuke Miyake, Aya Shinomiya, Yuka Yamamoto, Yoshihiro Nishiyama, Takashi Tamiya

**Affiliations:** 1 Department of Neurological Surgery, Faculty of Medicine, Kagawa University, Miki-cho, Kagawa Japan; 2 Department of Neurological Surgery, Kagawa Rehabilitation Hospital, 1114 Tamura-cho, Takamatsu-shi, Kagawa 761-8057 Japan; 3 Department of Diagnostic Radiology, Faculty of Medicine, Kagawa University, Miki-cho, Kagawa Japan

**Keywords:** Chromosome 1p and 19q codeletion, [^18^F]-Fluoromisonidazole (FMISO), [^18^F]-Fluoro-2-deoxy-d-glucose (FDG), Glioma, *IDH* mutation, Hypoxia, Glucose metabolism, PET/CT

## Abstract

**Background:**

Tumor hypoxia and glycolysis have been recognized as determinant factors characterizing tumor aggressiveness in malignant gliomas. To clarify in vivo hypoxia and glucose metabolism in relation to *isocitrate dehydrogenase* (*IDH*) mutation and chromosome 1p and 19q (1p/19q) codeletion status, we retrospectively analyzed hypoxia as assessed by positron emission tomography (PET) with [^18^F]-fluoromisonidazole (FMISO) and glucose metabolism as assessed by PET with [^18^F]-fluoro-2-deoxy-d-glucose (FDG) in newly diagnosed malignant gliomas.

**Methods:**

In total, 87 patients with newly diagnosed supratentorial malignant (WHO grade III and IV) gliomas were enrolled in this study. They underwent PET studies with FMISO and FDG before surgery. The molecular features and histopathological diagnoses based on the 2016 WHO classification were determined using surgical specimens. Maximal tumor-to-normal ratio (TNR) was calculated for FDG PET, and maximal tumor-to-blood SUV ratio (TBR) was calculated for FMISO PET. The PET uptake values in relation to *IDH* mutation and 1p/19q codeletion status were statistically analyzed.

**Results:**

In all tumors and malignant astrocytomas, the median FMISO TBR in *IDH*-wildtype tumors was significantly higher than that in *IDH*-mutant tumors (*P* < 0.001 and *P* < 0.01, respectively). In receiver operating characteristic (ROC) analysis, the area under the curve showed that the sensitivity for the discrimination was moderate (0.7–0.8) and the specificity was low (0.65–0.68). In the same population, the median FDG TNR in *IDH*-wildtype tumors tended to be higher than that in *IDH*-mutant tumors, but the difference was not statistically significant. In WHO grade III anaplastic astrocytomas, there were no significant differences in median FMISO TBR or FDG TNR between *IDH*-mutant and *IDH*-wildtype tumors. In *IDH*-mutant WHO grade III anaplastic gliomas, there were no significant differences in median FMISO TBR or FDG TNR between anaplastic astrocytomas and anaplastic oligodendrogliomas.

**Conclusions:**

Tumor hypoxia as assessed by FMISO PET was informative for prediction of the *IDH* mutation status in newly diagnosed malignant gliomas. However, the accuracy of the discrimination was not satisfactory for clinical application. On the other hand, glucose metabolism as assessed by FDG PET could not differentiate the *IDH*-mutant status. Moreover, PET studies using FMISO and FDG could not predict *IDH* mutation and 1p/19q codeletion status in WHO grade III tumors.

## Introduction

Tumor hypoxia and accelerated glycolysis have been recognized as determinant factors characterizing tumor aggressiveness [1, 2]. Tumor hypoxia is caused by the rapid depletion of oxygen that occurs with aberrant tumor cell proliferation and temporary cessation of blood flow due to disorganized vasculatures in malignant gliomas [2]. Hypoxia promotes neovascularization through a variety of molecular signals [3] and is associated with the propagation and progression of malignant gliomas [4]. One of the main early cellular events evoked upon exposure to hypoxia is the activation of hypoxia-inducible factor 1 (HIF-1) [5], an oxygen-regulated transcription factor that mediates the adaptation of cells to decreased oxygen supply. On the other hand, accumulating studies have demonstrated that accelerated aerobic glycolysis, also known as the Warburg effect, participates in the clinical aggressiveness of tumors including gliomas [6]. In contrast to normal cells, malignant cells use pyruvate for energy production instead of fluxing it into acetyl-CoA in the tricarboxylic acid (TCA) cycle, even under normoxic conditions. This effect is an adapted metabolic phenotype in cancer. The rate of glucose metabolism through aerobic glycolysis is higher, so the production of lactate from glucose occurs much faster than the complete oxidation of glucose in the TCA cycle at the expense of inefficient ATP production [7]. As a result, cancer cells overexpress glucose transporters to compensate for their high energy demand with their abnormally high rate of glucose uptake [6].

The updated 2016 edition of the World Health Organization (WHO) classification of tumors of the central nervous system (CNS) uses molecular parameters and histology to define the main tumor categories [8]. Among several molecular parameters, *isocitrate dehydrogenase* (*IDH*) mutation and chromosome 1p and 19q (1p/19q) codeletion status have pivotal roles in the characterization of gliomas and have been integrated in the new classification. Mutations of the enzyme cytosolic *IDH* in gliomas have drawn particular attention in the field of neuro-oncology in recent years [9]. Postoperative immunohistochemistry and sequencing have been commonly used to detect these molecular profiles. However, in some patients, tumor localization hinders ample sample collection for pathological assessment. Given that different *IDH* mutation statuses need different treatment strategies and exhibit different prognoses in gliomas [8, 10], it is essential to predict the *IDH* mutation status preoperatively and even in patients not undergoing surgery.

A recent study showed that *IDH*-mutant cells shift glucose metabolism from glycolysis to the TCA cycle [11, 12]. In addition, several clinical studies demonstrated that the glucose uptake of *IDH*-mutant gliomas was significantly lower than that of *IDH*-wildtype gliomas [13–15]. Recently, an exploratory study using oxygen-sensitive molecular MRI revealed that *IDH*-mutant gliomas exhibit lower hypoxia and acidity compared with *IDH*-wildtype gliomas [16]. Additionally, a study by Grassian et al. observed increased oxidative TCA metabolism and reduced tumor growth rates in *IDH*-mutant glioma cells under hypoxic conditions, suggesting that *IDH*-mutant gliomas prefer a more oxygenated microenvironment for cell proliferation [17]. Tumor hypoxia and glycolysis highlight an emerging theme in human malignant glioma, as they are major resistance factors responsible for the failure of chemo- and radiotherapy in malignant gliomas [6, 18].

Based on these studies, we hypothesized that *IDH*-mutant gliomas would exhibit both less hypoxia and lower glucose consumption compared with *IDH*-wildtype gliomas. To clarify in vivo hypoxia and glucose metabolism in relation to *IDH* mutation and 1p/19q codeletion status, we retrospectively analyzed the hypoxia as assessed by positron emission tomography (PET) with [^18^F]-fluoromisonidazole (FMISO) and glucose metabolism as assessed by PET with [^18^F]-fluoro-2-deoxy-d-glucose (FDG) in newly diagnosed malignant (WHO grade III and IV) gliomas.

## Materials and methods

### Patients

From April 2013 to March 2019, 87 patients with newly diagnosed supratentorial malignant gliomas (45 men and 42 women; median age 64 years, range 26–86 years) were retrospectively enrolled in this study. All patients underwent routine magnetic resonance imaging (MRI) examinations including T2-weighted fluid-attenuated inversion recovery (FLAIR) and gadolinium (Gd)-enhanced T1-weighted (T1 + Gd) sequences. Both PET studies were performed within a short period before surgery. Histopathology including immunohistochemistry (IHC) was performed on tissue specimens obtained by biopsy or resection using a multimodal navigation system (StealthStation®, Medtronic Sofamor Danek Co., Ltd., Tokyo, Japan) by integrating PET/CT images with anatomical MR images. The highest PET tracer uptake area was selected for tumor sampling. All gliomas were classified using the 2016 WHO classification. The presence of *IDH1* mutation was assessed by IHC to detect *IDH1* R132H (codon 132 of the *IDH1* gene) protein expression using a monoclonal antibody (clone H09, 1:50; Dianova, Hamburg, Germany). In cases where IHC was negative, *IDH1* and *IDH2* (R172) were directly sequenced using the Sanger method. The presence or absence of 1p/19q codeletion was determined by fluorescence in situ hybridization analysis. The 1p/19q codeletion status is another important genetic alteration in the 2016 WHO classification. The presence of both the *IDH* mutation and 1p/19q codeletion characterizes oligodendrogliomas, whereas the presence of the *IHD* mutation without 1p/19q codeletion is indicative of astrocytoma [8].

The patients’ characteristics, including histopathological and genetic diagnoses according to the 2016 WHO classification, are summarized in Table 1: 18 patients had grade III astrocytoma (anaplastic astrocytoma, AA), 11 patients had grade III oligodendroglioma (anaplastic oligodendroglioma, AO), and 58 patients had grade IV astrocytoma (glioblastoma, GBM). *IDH* mutation was found in 21 tumors (24.1%; 18 grade III, 3 grade IV), and 66 tumors were classified as *IDH*-wildtype tumors (75.9%; 11 grade III, 55 grade IV).

**Table 1:** Summary of 87 patients

Characteristics	Value
*Age (years), median (range)*	64 (26–86)
*Sex, n (%)*	
Male	45 (51.7)
Female	42 (48.3)
*WHO 2016 grade, n (%)*	
III	29 (33.3)
IV	58 (66.7)
*Histology, n (%)*	
Astrocytoma	76
AA	18 (20.7)
IDH-mut	7
IDH-wt	11
GBM	58 (66.7)
IDH-mut	3
IDH-wt	55
Oligodendroglioma	11
AO, IDH-mut & codel	11 (12.6)
*IDH mutation, n (%)*	
Mutant	21 (24.1)
Wildtype	66 (75.9)

Codel = 1p/19q codeleted

### Radiotracer synthesis

The clinical use of FMISO as a PET tracer was approved by the Kagawa University Faculty of Medicine Human Subjects Ethical Committees, and informed consent was obtained from all patients with written consent received before the PET examination. FMISO was prepared by a modified method of Oh et al. [19]. The radiochemical purity of the produced FMISO was greater than 99%. FDG was produced by proton bombardment of ^18^O-enriched water by the method of Toorongian et al. [20], with some modifications. The radiochemical purity of the produced FDG was greater than 95%.

### PET/CT imaging

PET studies were performed using an ECAT EXACT HR + scanner (Siemens/CTI, Knoxville, TN, USA) or a Biograph mCT PET/CT scanner (Siemens/CTI, Knoxville, TN, USA). Attenuation correction was performed using ^68^Ge rod sources rotating around the head or CT maps. The interval between FMISO and FDG PET scans was 2 weeks at most. No treatments were started until both PET scans had been completed. For FMISO PET, no special dietary instructions were given to the patients. Regional emission scans were acquired for 10 min, beginning 120 min after an intravenous bolus injection of FMISO (262 ± 54.3 MBq). For FDG PET, patients were instructed to fast for at least 6 h before imaging, and serum glucose levels were analyzed before the injection of FDG and were within normal levels. Each patient received an intravenous injection of FDG (235 ± 48.8 MBq). After 45–60 min, regional emission images of the brain were obtained for 10 min. Images were acquired with the patients resting in the supine position with their eyes closed. The PET data were acquired in three-dimensional mode and were reconstructed with the baseline ordered-subsets expectation maximization (OSEM) algorithm. For FMISO PET, a venous blood sample was obtained during emission tomography. A 1-mL whole blood sample was counted in a calibrated gamma well counter (ALOKA, Tokyo, Japan). Blood activity of the sample was expressed as MBq/mL of decay corrected to the time of injection.

### PET data analysis

Experienced nuclear medicine physicians analyzed the PET data. They were blinded to the results of the histopathological and immunohistochemical analyses.

The FMISO PET images were scaled to the venous blood concentration of FMISO activity to produce tumor-to-blood (T/B) values. This allowed for a pixel-by-pixel calculation of T/B activity ratios for all image planes. The maximum tumor-to-blood ratio (TBR) was calculated as the representative value for each tumor.

For FDG PET analysis, the region of interest (ROI) was outlined within areas of the hypermetabolic region in each slice of the SUV image. The SUV_max_ was calculated accordingly and considered the representative value for each tumor. The maximal tumor-to-normal ratio (TNR) was calculated by dividing the tumor SUV_max_ by the mean SUV of the normal brain parenchyma (usually contralateral normal brain excluding the ventricles). The PET and MRI datasets were transferred to a Linux workstation, and coregistration was performed using Dr. View/Linux (AJS, Tokyo, Japan).

### Statistical analysis

The Mann–Whitney *U* test was used to compare the TBR values for FMISO and TNR values for FDG in relation to *IDH* mutation and 1p/19q codeletion status. ROC analysis was performed to compare the diagnostic usefulness of the TBR for FMISO in predicting *IDH* mutation status. We determined the cutoff value at which the point is closest to (1, 1) corner in the ROC plane. All statistical analyses were performed using EZR version 1.40 (Saitama Medical Center, Jichi Medical University, Saitama, Japan). A *P* value less than 0.05 was considered statistically significant.

## Results

### PET uptake values in relation to *IDH* mutation status in all tumors

**Fig. 1:**
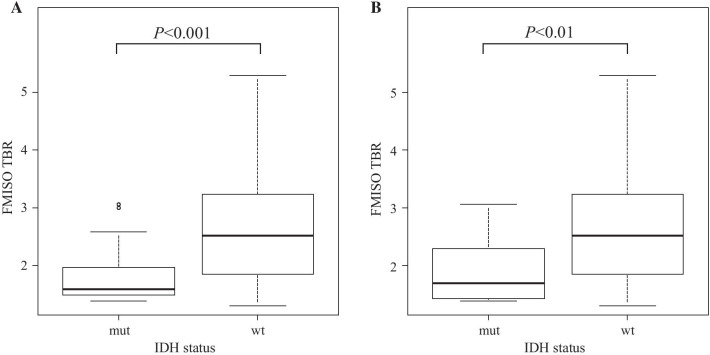
**a** Box plots showing the FMISO TBR in relation to *IDH1* mutation status in all gliomas (*n* = 87). The FMISO uptake in *IDH1*-wildtype (wt) tumors was significantly higher than that in *IDH1*-mutant (mut) tumors (*P* < 0.001). **b** Box plots showing the FMISO TBR in relation to *IDH1* mutation status in malignant astrocytomas (*n* = 76; 18 AAs and 58 GBMs). The FMISO uptake in *IDH1*-wildtype (wt) tumors was significantly higher than that in *IDH1*-mutant (mut) tumors (*P* < 0.01)

**Fig. 2:**
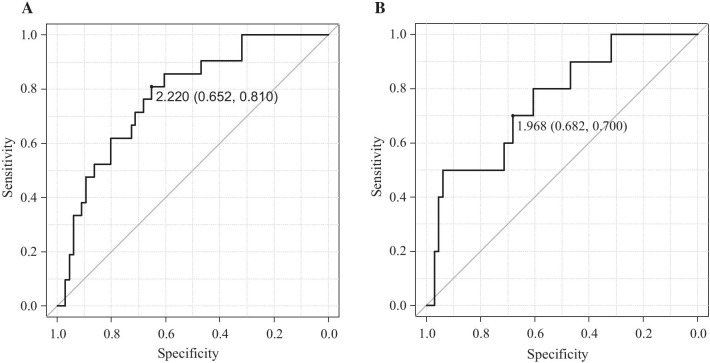
**a** ROC curve of FMISO PET for differentiating *IDH1*-wildtype tumors from *IDH1*-mutant tumors in all malignant gliomas (*n* = 87). The area under the curve (AUC) of the FMISO TBR was 0.777 (95% CI 0.669–0.885). When the cutoff value for the FMISO TBR in the ROC curve was set at 2.22, the sensitivity for the differential diagnosis was 81.0%, and the specificity was 65.2%. **b** ROC curve of FMISO PET for differentiating *IDH1*-wildtype tumors from *IDH1*-mutant tumors in malignant astrocytomas (*n* = 76; 18 AAs and 58 GBMs). The AUC of the FMISO TBR was 0.758 (95% CI 0.598–0.917). When the cutoff value for the FMISO TBR in the ROC curve was set at 1.968, the sensitivity for the differential diagnosis was 70%, and the specificity was 68.2%

In all tumors, the median FMISO TBRs for *IDH*-mutant and *IDH*-wildtype tumors were 1.60 (IQR 1.49–1.97) and 2.52 (1.86–3.21), respectively (Fig. 1a and Table 2). The median FMISO TBR for *IDH*-wildtype tumors was significantly higher than that for *IDH*-mutant tumors (*P* < 0.001). The ROC analysis performed to differentiate *IDH*-mutant tumors from *IDH*-wildtype tumors showed that the area under the curve (AUC) for the FMISO TBR was 0.777 (95% CI 0.669–0.885; Fig. 2a). When the cutoff value for the FMISO TBR in the ROC curve was set at 2.22, the sensitivity for the differential diagnosis was 81.0%, and the specificity was 65.2%.

**Table 2:** Summary of FMISO TBRs and FDG TNRs

	FMISO TBR, median (IQR)	FDG TNR, median (IQR)
*IDH status (n* = *87)*		
Mutant (*n* = 21)	1.60 (1.49–1.97)	2.17 (1.50–3.45)
Wildtype (*n* = 66)	2.52 (1.86–3.21)	2.55 (1.97–4.35)
*Histology and subtype*		
AA (*n* = 18)		
IDH-mut (*n* = 7)	1.47 (1.42–1.70)	1.50 (1.23–2.55)
IDH-wt (*n* = 11)	1.57 (1.46–1.86)	1.90 (1.54–2.39)
AO (*n* = 11)	1.60 (1.53–1.80)	2.21 (1.83–3.11)
AA + GBM (*n* = 76)		
IDH-mut (*n* = 10)	1.70 (1.44–2.21)	1.76 (1.31–3.62)
IDH-wt (*n* = 66)	2.52 (1.86–3.21)	2.55 (1.97–4.35)
GBM (*n* = 58)		
IDH-mut (*n* = 3)	2.58 (2.27–2.82)	3.80 (2.59–3.95)
IDH-wt (*n* = 55)	2.83 (2.24–3.45)	2.73 (2.10–4.41)

*IQR* interquartile range

The median FDG TNRs for *IDH*-mutant and *IDH*-wildtype tumors were 2.17 (IQR 1.50–3.45) and 2.55 (1.97–4.35), respectively (Table 2). The median FDG TNR for *IDH*-wildtype tumors tended to be higher than that for *IDH*-mutant tumors, but the difference did not reach statistical significance (*P* = 0.077).

### PET uptake values in relation to *IDH* mutation status in AAs and GBMs

In malignant astrocytomas (18 AAs and 58 GBMs) excluding AOs, the median FMISO TBRs for *IDH*-mutant and *IDH*-wildtype tumors were 1.70 (IQR 1.44–2.21) and 2.52 (1.86–3.21), respectively (Fig. 1b and Table 2). The median FMISO TBR for *IDH*-wildtype tumors was significantly higher than that for *IDH*-mutant tumors (*P* < 0.01). The ROC analysis performed to differentiate *IDH*-mutant tumors from *IDH*-wildtype tumors showed that the AUC for the FMISO TBR was 0.758 (95% CI 0.598–0.917; Fig. 2b). When the cutoff value for the FMISO TBR in the ROC curve was set at 1.968, the sensitivity for the differential diagnosis was 70%, and the specificity was 68.2%.

The median FDG TNRs for *IDH*-mutant and *IDH*-wildtype tumors were 1.76 (IQR 1.31–3.62) and 2.55 (1.97–4.35), respectively (Table 2). The median FDG TNR for *IDH*-wildtype tumors tended to be higher than that for *IDH*-mutant tumors, but the difference did not reach statistical significance (*P* = 0.105).

### PET uptake values in relation to *IDH *mutation and 1p/19q *codeletion* status in WHO grade III anaplastic tumors

We further analyzed the PET uptake values for WHO grade III anaplastic tumors (7 AAs with *IDH* mutation, 11 AAs without *IDH* mutation, and 11 AOs with 1p/19q codeletion and *IDH* mutation).

In WHO grade III AAs, the median FMISO TBRs for *IDH*-mutant and *IDH*-wildtype AAs were 1.47 (IQR 1.42–1.70) and 1.57 (IQR 1.46–1.86), respectively (Table 2). There was no significant difference in FMISO TBR between the two groups. The median FDG TNRs for *IDH*-mutant and *IDH*-wildtype AAs were 1.50 (IQR 1.23–2.55) and 1.90 (IQR 1.54–2.39), respectively (Table 2). There was no significant difference in FDG TNR between the two groups.

In *IDH1*-mutant WHO grade III anaplastic gliomas, the median FMISO TBRs for AAs and AOs were 1.47 (IQR 1.42–1.70) and 1.60 (IQR 1.53–1.80), respectively (Table 2). There was no significant difference in FMISO TBR between the two groups. The median FDG TNRs for AAs and AOs were 1.50 (IQR 1.23–2.55) and 2.21 (IQR 1.83–3.11), respectively (Table 2). There was no significant difference in FDG TNR between the two groups.

### Illustrative cases

**Fig. 3:**
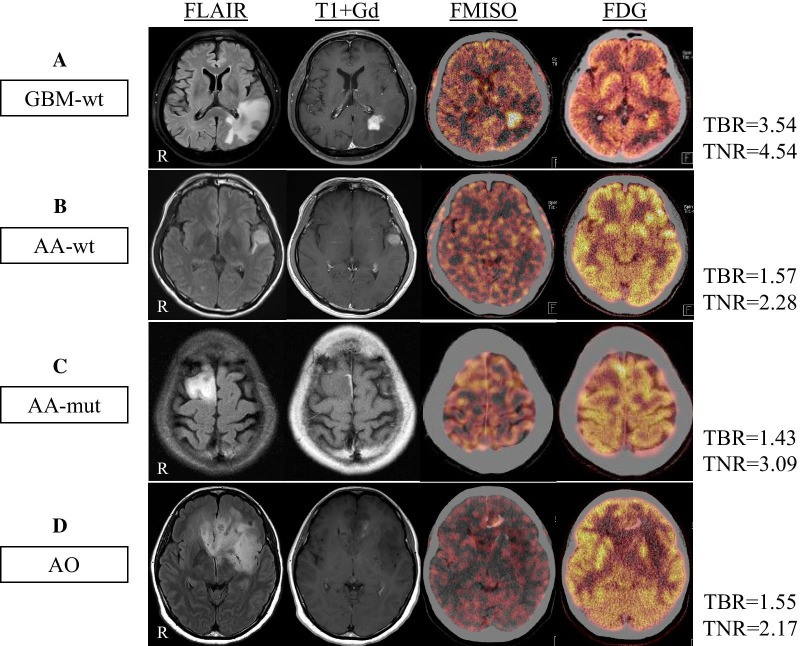
MRI (FLAIR and T1 + Gd) and PET images (FMISO and FDG) in representative glioma patients. **a** A 66-year-old male patient with GBM-wt showing high uptake of FMISO (TBR = 3.54) and FDG (TNR = 4.54). **b** A 36-year-old male with AA-wt showing slightly increased uptake of FMISO (TBR = 1.57) and FDG (TNR = 2.28). **c** A 31-year-old female with AA-mut showing slightly increased uptake of FMISO (TBR = 1.43) and high uptake of FDG (TNR = 3.09). **d** A 38-year-old female with AO showing slightly increased uptake of FMISO (TBR = 1.55) and FDG (TNR = 2.17)

Figure 3 shows representative MRI and FMISO and FDG PET images in each molecular genomic subtype.

## Discussion

In the present study, it was clearly demonstrated that hypoxia as assessed by FMISO PET was significantly higher in *IDH*-wildtype tumors than in *IDH*-mutant tumors in newly diagnosed malignant (WHO grade III and IV) gliomas. A significant difference was also observed in malignant astrocytomas (AAs and GBMs) excluding AOs. These results are hardly surprising, as 58 of 87 patients (66.7%) enrolled in the present study had GBMs and 55 of the 58 GBMs (94.8%) were *IDH*-wildtype tumors. Previous studies have shown that FMISO uptake is significantly higher in GBMs compared with lower-grade tumors and that FMISO does not highly accumulate in most non-GBM patients [21, 22]. On the other hand, glucose metabolism as assessed by FDG PET in *IDH*-wildtype tumors tended to be higher than that in *IDH*-mutant tumors in the present study, but the difference did not reach statistical significance. This result was not in agreement with previous studies, in which the FDG uptake of *IDH*-wildtype tumors was significantly higher than that of *IDH*-mutant tumors [13–15]. This could be because the previous studies included all grades of diffuse gliomas (WHO grade II–IV), but the present study examined FDG PET only in malignant tumors (WHO grade III and IV). The great majority of WHO grade II tumors fall into the *IDH*-mutant category, and *IDH*-wildtype is an uncommon diagnosis [8]. FDG PET was suggested to be useful for imaging brain tumors because of increased glucose metabolism in high-grade tumors as well as positive correlation between the glycolysis rate and malignancy [23]. However, the diagnostic accuracy of FDG PET is hampered by the high physiologic glucose metabolism in surrounding brain areas and poor reliability in predicting the neoplastic nature of the lesions [24]. Moreover, studies have revealed that FDG PET has low sensitivity in differentiating low-grade tumors from high-grade tumors [25].

Anaplastic gliomas, which are defined as WHO grade III tumors, are considered to be malignant because of the short survival of patients with them. Furthermore, the prognosis of these patients varies and depends on the molecular genomic subtype. Recent genome-wide mutational analyses revealed that this clinical heterogeneity was largely related to *IDH* mutation and 1p/19q codeletion status [26]. AOs typically present with 1p/19q-codeletion combined with *IDH* mutation and have the best prognosis of all WHO grade III tumors. AA can be further differentiated into subgroups based on the *IDH* mutation status. The prognosis of *IDH*-mutant AA is intermediate, whereas *IDH*-wildtype AA is linked to a poor prognosis and bears many similarities to GBM [26]. A previous study reported that the prognostic role of *IDH* mutation status was prominent, especially in AAs, and the median overall survival was 65 months in patients with *IDH* mutations and 20 months in those without mutations [9]. Therefore, we further analyzed the FMISO and FDG uptakes in WHO grade III tumors to evaluate the actual relationships of *IDH* mutation and 1p/19q codeletion status with in vivo hypoxia and glucose metabolism. Although the number of tumors was small, hypoxia and glucose metabolism as assessed by PET were not significantly different between *IDH*-wildtype and *IDH*-mutant tumors in newly diagnosed AAs. This result was not in agreement with a previous study in which Takei et al. examined WHO grade III AAs and showed that *IDH*-wildtype tumors had significantly higher FDG uptakes compared with *IDH*-mutant tumors [14]. They examined the *IDH* mutation status using only IHC analysis, whereas we examined it more accurately using both IHC analysis and direct DNA sequencing. There are some reports describing a discrepancy between the results of IHC analysis and direct DNA sequencing [27]. The present study also showed that in vivo hypoxia and glucose metabolism as assessed by FMISO and FDG PET were not significantly different between AOs and AAs in *IDH1*-mutant WHO grade III tumors. This was in agreement with a previous study, in which Takei et al. showed that there was no significant difference in FDG uptake between *IDH*-mutant AOs and *IDH*-mutant AAs [14]. The overall survival for AOs has been reported to be significantly longer than that for AAs [28]. These results raise a question about the diagnostic and prognostic accuracy of in vivo hypoxic and glycolytic biomarkers assessed by FMISO and FDG PET and molecular biomarkers such as *IDH* mutation and 1p/19q codeletion status in WHO grade III tumors.

In the present study, we used FMISO and FDG PET with the assumption that these tracers can detect hypoxia and glucose metabolism accurately in human brain tumors. However, they have specific characteristics requiring considerable attention for detecting hypoxia and evaluating glycolysis. For FMISO, the threshold of oxygen partial pressure that determines whether FMISO is accumulated or extracted is generally believed to be approximately 10 mmHg [29]. FMISO accumulates only in severely hypoxic tissue, and these hypoxic conditions might be to induce tissue necrosis. Moreover, we obtained a static image 120 min after intravenous bolus injection of FMISO. There are several reports that a dynamic scan mode and the time activity curve of the tumors can evaluate hypoxia and other characteristics of the tumor biology better than using the static scan [30]. Later, ^62^Cu-diacetyl-bis(N4-methylthiosemicarbazone) (Cu-ATSM) has been proposed as a promising hypoxic tracer for PET [31]. In vivo studies have demonstrated that tissue Cu-ATSM uptake is dependent on the oxygen concentration and is retained in hypoxic cells at a higher level than FMISO [32]. For FDG, a previous study reported that it was successfully employed in the evaluation of cancer patients under the assumption that an increase in aerobic glycolysis would be reflected by an increase in the total glucose consumption of the tissue, although false-negative results do occur [33]. The degree to which these false-negative results would reflect a discrepancy between glucose metabolism as determined by conventional FDG PET scan and actual aerobic glycolysis is not well known. Recently, an ^18^F-labeled specific radiotracer, DASA-23, has been synthesized for evaluating in vivo pyruvate kinase 2 expression, which catalyzes the final and rate-limiting step in tumor glycolysis [34].

The present study has several limitations. First, this study was a retrospective nature based on postoperative histopathological diagnosis. In a clinical setting, preoperative prediction of *IDH* mutation status in all grade gliomas is more important. In our hospital, all of the patients suspected having glioma were also examined using [^11^C]-methionine (MET) and [^18^F]-fluorothymidine (FLT). Recently, we have reported the tracer uptake in grade III gliomas was significantly higher than that in grade II gliomas in MET and FLT PET in *IDH*-mutant tumors [35]. We also demonstrated that there was a significant correlation between FMISO uptake and glioma grade, with all low-grade gliomas (grade I and II) demonstrating no hypoxia and all high-grade gliomas (grade III and IV) showing hypoxia in newly diagnosed gliomas [22]. Therefore, we could accurately discriminate malignant gliomas from low-grade gliomas using several PET tracers preoperatively. Second, we could not statistically compare FMISO and FDG uptake values between *IDH*-wildtype and *IDH*-mutant tumors in newly diagnosed GBMs because there were only 3 GBMs with *IDH* mutation. The incidence of *IHD*-mutant GBM is rare (less than 10%) in the general population [8]. Third, we could not perform volumetric analyses between *IDH*-wildtype and *IDH*-mutant tumors. According to previous reports, both the intensity and volume of glucose metabolism and hypoxic burden are associated with prognosis in GBM patients [36]. The metabolically active hypoxic volume can be segmented as the volume showing both FMISO and FDG uptakes [36]. In the present study, we investigated PET images using two types of PET scanner (ECAT EXACT HR+ and Biograph mCT PET/CT). Therefore, the differences in spatial resolution and image contrast could have affected delineation of FMISO- and FDG-positive lesions. Finally, the present study was retrospective in nature, and no follow-up data were evaluated. Therefore, the findings need to be analyzed in relation to prognosis with a long follow-up period.

## Conclusion

The present study demonstrated that tumor hypoxia as assessed by FMISO PET in *IDH*-wildtype tumors was significantly higher than that in *IDH*-mutant tumors and that the FMISO uptake value was able to predict the *IDH* mutation status in newly diagnosed malignant gliomas. However, the sensitivity for the discrimination was moderate (0.7–0.8), and the specificity was low (0.65–0.68) in ROC analysis. On the other hand, glucose metabolism as assessed by FDG PET could not differentiate the *IDH*-mutant status. Moreover, conventional PET studies using FMISO and FDG could not predict the *IDH* mutation and 1p/19q codeletion status in WHO grade III tumors. The absence of hypoxic and glycolytic signatures in relation to *IDH* mutation status might reflect specific characteristics of FMISO and FDG for detecting hypoxia and glucose metabolism in in vivo PET study. Further research using alternative or advanced radiotracers for hypoxic and glycolytic imaging is warranted to precisely predict molecular biomarkers, especially for the *IDH1* mutation status.

## Data Availability

Due to the sensitive nature of human participant information, all datasets used during the current study are not publicly open but are available upon reasonable request by contacting the corresponding author.

## Abbreviations

AA: anaplastic astrocytoma
AO: anaplastic oligodendroglioma
ATP: adenosine triphosphate
AUC: area under the curve
CNS: central nervous system
CT: computed tomography
EZR: easy R
FDG: ^18^F-fluoro-2-deoxy-d-glucose
FLAIR: fluid-attenuated inversion recovery
FMISO: ^18^F-fluoromisonidazole
FOV: field of view
FWHM: full-width at half maximum
GBM: glioblastoma multiforme
Gd: gadolinium
HIF-1: hypoxia-inducible factor 1
IDH: isocitrate dehydrogenase
IHC: immunohistochemistry
IQR: interquartile range
MBq: megabecquerel
MRI: magnetic resonance imaging
OSEM: ordered subset expectation maximization
PET: positron emission tomography
ROC: receiver operating characteristic
ROI: region of interest
SUV: standardized uptake value
TBR: tumor-to-blood ratio
TCA: tricarboxylic acid
TNR: tumor-to-normal ratio
WHO: World Health Organization
